# Towards understanding the evolution and functional diversification of DNA-containing plant organelles

**DOI:** 10.12688/f1000research.7915.1

**Published:** 2016-03-11

**Authors:** Dario Leister

**Affiliations:** 1 Plant Molecular Biology, Department Biology I, Ludwig-Maximilians-Universität, Planegg-Martinsried, 82152, Germany; 2 Copenhagen Plant Science Center (CPSC), University of Copenhagen, Thorvaldsensvej 40, 1871 Frederiksberg C, Denmark

**Keywords:** mitochondria, nuclear mitochondrial DNA, nuclear plastid DNA, phylogenomics, proteome, chloroplast

## Abstract

Plastids and mitochondria derive from prokaryotic symbionts that lost most of their genes after the establishment of endosymbiosis. In consequence, relatively few of the thousands of different proteins in these organelles are actually encoded there. Most are now specified by nuclear genes. The most direct way to reconstruct the evolutionary history of plastids and mitochondria is to sequence and analyze their relatively small genomes. However, understanding the functional diversification of these organelles requires the identification of their complete protein repertoires – which is the ultimate goal of organellar proteomics. In the meantime, judicious combination of proteomics-based data with analyses of nuclear genes that include interspecies comparisons and/or predictions of subcellular location is the method of choice. Such genome-wide approaches can now make use of the entire sequences of plant nuclear genomes that have emerged since 2000. Here I review the results of these attempts to reconstruct the evolution and functions of plant DNA-containing organelles, focusing in particular on data from nuclear genomes. In addition, I discuss proteomic approaches to the direct identification of organellar proteins and briefly refer to ongoing research on non-coding nuclear DNAs of organellar origin (specifically, nuclear mitochondrial DNA and nuclear plastid DNA).

## Introduction

The progenitors of the non-nuclear DNA-containing organelles of plants – plastids and mitochondria – were originally acquired as cyanobacterial and proteobacterial endosymbionts, respectively (reviewed in 1– 4). As they co-evolved with their host cells, the original endosymbionts lost most of their genetic repertoires, either definitively or through transfer to the host’s nuclear genome. In parallel, having picked up suitable signal sequences, the products of many nuclear genes of endosymbiotic origin were re-routed back to their original compartment, together with new nucleus-encoded proteins, via intracellular trafficking routes ^5– 10^. As a result, complex organellar proteomes now consist of several thousand different proteins – similar in the total number of different proteins, though less so in composition, to the proteomes of their closest prokaryotic relatives.

To reconstruct the evolutionary history of plastids and mitochondria, analysis of the coding regions of the relatively small residual organellar genomes is the most straightforward approach and has helped us to understand such post-endosymbiotic events as gene loss, nuclear transfer of organellar genes, and organelle evolution in general. Moreover, coding and non-coding organellar DNA can be used as a barcode to elucidate relationships between species ^11^. However, to approach the diversification of the functions of organelles in a comprehensive way, ideally their entire proteomes must be identified. Since only partial organellar proteomes can be identified by proteomics, a powerful complementation (or alternative when proteomics is impracticable) is to bioinformatically analyze the corresponding complement of their nuclear genes. This is a formidable challenge and only became feasible when entire nuclear genome sequences of plant species became available. In this review, I summarize genome-wide approaches to the definition of the protein contents of organelles, as well as interspecies comparisons of entire organellar and nuclear genomes (phylogenomics) that have contributed to our understanding of the evolution of organellar proteomes. In addition, I will discuss selected proteomic analyses of organellar proteins and briefly introduce non-coding nuclear DNA sequences of organellar origin as “by-products” of organelle evolution.

## Phylogenomic approaches employing organellar DNA sequences

Traditionally, plant molecular phylogenetics has involved amplifying, sequencing, and analyzing one or a few genes from many species. Alternatively, entire genomes can be sequenced and analyzed (phylogenomics), providing much larger amounts of data per taxon but often for a smaller number of species ^12^. Nowadays, ample sequence information on DNA-containing organelles is available, i.e. the ChloroMitoSSRDB database currently provides access to 2161 organellar genomes (1982 mitochondrial and 179 chloroplast genomes) ^13^. Because of their small size, mitochondrial and plastid genomes from different species were the first to be analyzed by phylogenomic approaches. The outcome of such interspecific comparisons turns out to be highly dependent on the sample size. This is illustrated by two pioneering studies performed 4 years apart by the same group with a view to reconstructing plastid evolution ^14, 15^. In these analyses, 9 and 15 plastid genomes, respectively, were compared, and a total of 210 and 274 different protein-coding plastid genes were identified. Of these, 45 and 44, respectively, were found in all plastid genomes in the respective set, while 44 and 117 proteins found in at least one plastid genome had nucleus-encoded counterparts in other species ^14, 15^.

Whereas the first complete plastid DNA (ptDNA) sequences were published 30 years ago ^16, 17^, it took a while longer for the first two plant mitochondrial genomes to be sequenced ^18, 19^, primarily because plant mitochondrial DNAs (mtDNAs) are much larger (e.g. ~370 kbps: *Arabidopsis thaliana*) than animal mtDNAs ^20, 21^ or ptDNAs (e.g. ~150 kbps for *A. thaliana*). Because mitochondria are common to all eukaryotes, their phylogenetic and phylogenomic analysis markedly contributed to the elucidation of the deep branching order of all eukaryotes, including protist, fungal, animal, and plant lineages (reviewed by 22). However, in the mitochondria of land plants, frequent genomic rearrangements, the incorporation of foreign DNA from nuclear and chloroplast genomes, and peculiarities of gene expression – most notably RNA editing and *trans*-splicing – are significantly more prominent than in chloroplasts (reviewed by 23). Furthermore, the physical organization of plant mtDNAs includes a mixture of linear, circular, and branched structures, resulting from homologous recombination – which appears to be an essential characteristic of plant mitochondrial genetic processes, both in shaping and in maintaining the genome (reviewed by 24).

## Estimating organellar proteomes

### Plastids

The first publication predicting the size and evolutionary origin of the chloroplast proteome encoded in the (at that time incompletely sequenced) nuclear genome of the flowering plant *A. thaliana* identified the genes for chloroplast proteins based on the fact that their predicted products bore chloroplast transit peptides (cTPs) ^25^ ( Table 1). The study predicted between 1900 and 2500 nucleus-encoded chloroplast proteins, of which a minimum of 35% derived from the cyanobacterial ancestor. In the entire *A. thaliana* genome sequence, 3574 (14.0%) genes coding for chloroplast proteins were identified by a prediction program ^26^, but the total number of cTPs obtained was not corrected for the expected numbers of false positives and negatives. Such genome-wide predictions have been repeated several times, employing different versions (with continuously improved annotation) of the Arabidopsis genome and different types or combinations of predictors (see Table 1). Interspecies comparisons of the sets of predicted chloroplast proteins have also been performed. The first such comparison published, between Arabidopsis and rice, conservatively estimated that some 2100 ( *A. thaliana*) and 4800 ( *Oryza sativa*) proteins carried cTPs, and defined a subset of around 900 tentative chloroplast proteins, predominantly derived from the cyanobacterial endosymbiont and with functions mostly related to metabolism, energy, and transcription, that is shared by both species ^27^.

**Table 1.** Overview of organellar proteome size predictions and selected proteomics approaches in Arabidopsis. Note that for the predictor TPpred only the total number of 3194 Arabidopsis proteins with either chloroplast transit peptides (cTP) or mitochondrial transit peptides (mTP) was reported ^68^.

Approach	(Estimated) number	Reference
chloroplast		
cTP prediction (ChloroP) and correction for false positives/negatives	1900–2500	25
cTP prediction (TargetP)	3574	26
cTP prediction (TargetP)	3646	69
cTP prediction (TargetP) and correction for false positives/negatives	3130	31
cTP prediction (combination of predictors) and correction for false positives/negatives	2090	27
cTP prediction (Predotar)	1591	70
cTP prediction (TargetP)	4255	71
Mass spectrometry	690	72
Mass spectrometry + literature search	916	30
mitochondrion		
mTP prediction (TargetP)	2897	26
mTP prediction (TargetP) and correction for false positives/negatives	3135	31
mTP predictions (combinations of predictors) and correction for false positives/negatives	2957	73
mass spectrometry	416	28
mTP predictions (combination of predictors)	2955–4514	28
mTP prediction (Predotar)	1105	70

As outlined above and shown in Table 1, genome-wide cTP predictions vary markedly in their outcome, depending on the type or combination of predictors used, and their sensitivity and specificity. In fact, a detailed comparative analysis of the performance of five different predictors for subcellular targeting demonstrated a disappointingly small overlap between the outcomes of different predictions. Conversely, when all predicted proteins that had been identified by at least one of the programs were considered, far too many proteins were found to have been assigned to a specific compartment ^28^. This clearly shows that predictive models inevitably involve a trade-off. Tightly constrained models which pinpoint *only* proteins that are truly located in the respective compartment (i.e. with high specificity) will fail to detect all of the proteins actually localized there (many false negatives), whereas saturated predictions that identify *most* of the truly located proteins (i.e. with high sensitivity) will also turn up many proteins that are actually destined for other compartments (many false positives). Moreover, a subset of chloroplast proteins does not contain cTPs, either because these proteins are inserted in the outer membrane or because they employ another ER-dependent pathway for targeting and import into chloroplasts (reviewed by 9, 29) – although the latter fraction may well be quite small ^30^.

Instead of first predicting the entire set of chloroplast proteins and then analyzing their homology with proteins from other species (in particular cyanobacteria, to identify proteins derived from the original endosymbiont), one can do the reverse. In fact, a comparison of all *A. thaliana* proteins with those encoded in cyanobacterial genomes, other prokaryotic reference genomes, and yeast allowed its authors to extrapolate that ~4500 *A. thaliana* protein-coding genes had been acquired from the cyanobacterial ancestor of plastids ^15^ and the products of some 1300 should belong to the predicted chloroplast proteome of 3100 proteins ^31^. Since then, the identity of the ancient cyanobacterial endosymbiont that gave rise to all contemporary plastids was narrowed down to the progenitors of diazotrophic cyanobacterial lineages because the gene set possessed by their modern-day representatives shows the greatest similarity to that predicted for the plastid ancestor ^32^.

Interspecies comparisons of nuclear genomes that do not also consider the predicted subcellular location of their products do not in themselves permit reliable conclusions regarding plastid or mitochondrial functions. However, if the species to be compared are appropriately selected, indirect but important conclusions can be drawn with respect to the protein repertoires of organelles and their evolutionary diversification. An early phylogenomic study compared all protein-coding genes from only one plant species ( *A. thaliana*) with the genes from several animals, yeasts, and combined sets of bacteria and Archaea ^33^ and identified 3848 plant-specific proteins, of which about 27% were predicted to localize to chloroplasts or mitochondria. In 2007, the phylogenomic comparison of several photosynthetic eukaryotes with non-photosynthetic eukaryotes, cyanobacteria, non-photosynthetic eubacteria, and Archaea enabled researchers to define sets of plant proteins with plastid-associated functions without having to depend primarily on cTP predictions ^34^. The original set, the so-called *GreenCut*, comprised proteins that were conserved in the green algae *Chlamydomonas reinhardtii* and *Ostreococcus tauri*, the moss *Physcomitrella patens*, and the flowering plant *A. thaliana*, but were absent from non-photosynthetic organisms, and consisted of 349 proteins in *C. reinhardtii*. The more restrictive *PlastidCut* (with 90 proteins in *C. reinhardtii*) was made up of *GreenCut* proteins which were also conserved in one diatom and one red alga species. In 2011, a revised version of this analysis (with *GreenCut2* and *PlastidCut2*) became available, which was based on the analysis of a larger set of sequenced genomes ^35^. To qualify for *GreenCut2*, a protein must (i) have orthologs in *A. thaliana*, *P. patens*, *O. sativa*, *Populus trichocarpa*, *C. reinhardtii*, and one of the three *Ostreococcus* species with fully sequenced genomes and (ii) not have orthologs in a number of bacterial, fungal, and animal species. *GreenCut2* contained 597 *Chlamydomonas* (and 710 *Arabidopsis* orthologs due to gene duplications) and *PlastidCut2* covers 124 proteins in *C. reinhardtii*. A subset (84%) of the *PlastidCut2* proteins were experimentally localized to, or are predicted to be targeted to, the plastid and 52% of all *GreenCut2* proteins were experimentally localized to the chloroplast, implying that the majority of *GreenCut2* proteins are involved in plastid-specific functions. In line with this tentative assignment of plastid-related functions of *GreenCut* proteins, mutations in *GreenCut2* genes were sixfold over-represented in a screen for photosynthetic mutants in *C. reinhardtii* which used large-scale random insertional mutagenesis ^36^. However, it is intriguing that 6% (11%) of all *PlastidCut2* ( *GreenCut2*) proteins have been experimentally located in non-plastid sites.

Of the 597 *GreenCut2* proteins in *C. reinhardtii*, 105 were missing in at least one of the other green algae analyzed, and diatoms too display a reduced number of *GreenCut2* proteins. These findings suggest that (i) adaptation of green algae to specific environmental niches leads to genome specialization and/or reduction and (ii) several core plastid functions in the green lineage are either not essential or are performed by different pathways/processes in diatoms ^35^. In contrast, almost all *GreenCut2* proteins are conserved in the other plant genomes analyzed, suggesting that the *GreenCut2* proteins are especially relevant to, and representative of, all land plants of the green lineage ^35^. The suggestion that the extent of conservation of the *GreenCut2* inventory in a plant could serve as an indicator of a particular genome’s degree of specialization might be an oversimplification ^35^ – at least when applied to plastid proteome complexity – because one must take account of the fact that plants contain multiple types of plastids, such that each variant might be of similar complexity to those from green algae. Indeed, analysis of chloroplast differentiation in maize, rice, and tomato reveals remarkably dynamic changes in plastid proteomes during plant development. For instance, to accommodate C _4_ photosynthesis, maize chloroplasts differentiate along the developmental axis of the leaf blade, leading from an undifferentiated leaf base into highly specialized bundle sheath (BS) and mesophyll (M) types. Hundreds of proteins detected by proteomics show differential BS/M accumulation ^37^, displaying five developmental transitions ^38^. Analysis of etioplast-to-chloroplast differentiation in rice by proteomics has shown that etioplast metabolism is already primed to accommodate the metabolic changes that occur during the onset of photosynthesis, such that only minor metabolic network reconstruction and modification of enzyme levels occurs during the first phase of etioplast-to-chloroplast differentiation ^39^. During the chloroplast-to-chromoplast transition in tomato, proteomic analyses detected a strong decrease in the abundance of proteins required for the light reactions and carbohydrate metabolism, and an increase in terpenoid biosynthesis and stress-response proteins was noted ^40^.

### Mitochondria

The first phylogenomic approach that indirectly addressed the evolution of nuclear genes for mitochondrial proteins compared the nuclear protein-coding genes from *Saccharomyces cerevisiae* to the ones encoded by Bacteria and Archaea and found that about 75% of all yeast nuclear genes of tentatively prokaryotic origin are more similar to eubacterial than to archaebacterial homologs ^41^. This suggested that the common ancestor of eukaryotes may also have possessed a majority of eubacterial genes, though it is still unclear how many of these ultimately come from the ancestral mitochondrial genome. Subsequent analysis of a sample of 27 sequenced eukaryotic and 994 sequenced prokaryotic genomes identified a set of 571 genes that was presumed to be present in the common ancestor of eukaryotes, underscoring the archaebacterial (host) nature of the eukaryotic informational genes and the eubacterial (mitochondrial) nature of eukaryotic energy metabolism ^42^. A similar type of analysis indicated that gene transfer from bacteria to eukaryotes is episodic and coincides with major evolutionary transitions at the origin of chloroplasts and mitochondria ^43^.

Plant proteomics has also contributed to our understanding of the evolution of the mitochondrial proteome. For instance, a comparison of more than 347 mitochondrial proteins identified by proteomics in Chlamydomonas, with their homologs predicted from 354 sequenced genomes, indicated that Arabidopsis is the non-algal eukaryote most closely related to *C. reinhardtii* and that free-living α-proteobacteria belonging to the orders Rhizobiales ** and Rhodobacterales better reflect the gene content of the ancestor of the chlorophyte mitochondrion than parasitic α-proteobacteria do ^44^.

## Non-coding nuclear sequences of chloroplast or mitochondrial origin

The continuous transfer of genetic material from organelles to the nucleus can result in various outcomes with respect to the functionality of the resulting nuclear sequences (reviewed in 3, 45– 47): (i) rarely, but with high impact on gene evolution, functional genes are generated when the transferred open reading frame recruits appropriate elements for its expression. The product of the relocated gene can then be retargeted to its original compartment or acquire new subcellular locations and functions ^31^; (ii) Parts of the transferred organellar DNA can remain/become functional as material for new exons in other genes ^48^; (iii) In the vast majority of cases, the transferred organellar DNA becomes non-functional and accumulates mutations, resulting in the so-called nuclear mtDNA (NUMT) sequences (see e.g. 49– 55) and nuclear ptDNA (NUPT) sequences (see e.g. 56– 62). In plants, NUPTs and NUMTs can account for several hundred kbps of nuclear genomes, ranging from very small insertions to larger segments of mtDNA and/or ptDNA >100 kbps in length ^63^, which further facilitates study of the fate of alien DNA in the nuclear genome. 

## Conclusions

As yet, no single prediction program and no single proteomics experiment can accurately identify the full complement of proteins located in plastids or mitochondria. At least for model plants like *C. reinhardtii* and *A. thaliana*, a combination of predictions, large-scale fluorescence tagging, epitope tagging, proteomics of multiple subfractions of organelles, and studies of individual genes/proteins will remain the method of choice for identifying entire organelle proteomes. To this end, public and searchable databases with a web-accessible interface like SUBA3 ( http://suba3.plantenergy.uwa.edu.au/) ^64^ and PPDB ( http://ppdb.tc.cornell.edu/) ^65^ are now available, which integrate the results of various prediction programs of subcellular targeting proteins with large-scale proteomic datasets from cellular compartments. It needs to be remembered, however, that in the case of plants with distinct plastid variants, prediction programs will have their limitations. Here, only proteomics can reliably discriminate the diverse proteomes in the several differentiation types of plastids.

Evolutionary trees obtained by phylogenomic analyses have changed our perspective on the origin of eukaryotes by supporting hypotheses which postulate that the mitochondrial endosymbiont was acquired by an archaeon, thus placing eukaryotes within the Archaea. Therefore, phylogenomic analyses provided support for only two primary domains of life – Archaea and Bacteria – and eukaryotes arose through partnership between them (reviewed by 66). Moreover, the outcomes of phylogenomic analyses also strikingly illustrate the concept of “evolutionary tinkering” ^67^. The nucleus can recruit novel exons even from “junk DNA” derived from plastids and mitochondria, and genes from cyanobacteria or proteobacteria now code in plants for many proteins that are not in their original compartment but have ended up elsewhere in the cell.
